# A Kuramoto model of self-other integration across interpersonal synchronization strategies

**DOI:** 10.1371/journal.pcbi.1007422

**Published:** 2019-10-16

**Authors:** Ole Adrian Heggli, Joana Cabral, Ivana Konvalinka, Peter Vuust, Morten L. Kringelbach

**Affiliations:** 1 Center for Music in the Brain, Aarhus University & The Royal Academy of Music Aarhus/Aalborg, Aarhus, Denmark; 2 Life and Health Sciences Research Institute (ICVS), School of Medicine, University of Minho, Braga, Portugal; 3 Department of Psychiatry, University of Oxford, Oxford, United Kingdom; 4 SINe Lab, Section for Cognitive Systems, DTU Compute, Technical University of Denmark, Kongens Lyngby, Denmark; MIT, UNITED STATES

## Abstract

Human social behaviour is complex, and the biological and neural mechanisms underpinning it remain debated. A particularly interesting social phenomenon is our ability and tendency to fall into synchronization with other humans. Our ability to coordinate actions and goals relies on the ability to distinguish between and integrate self and other, which when impaired can lead to devastating consequences. Interpersonal synchronization has been a widely used framework for studying action coordination and self-other integration, showing that even in simple interactions, such as joint finger tapping, complex interpersonal dynamics emerge. Here we propose a computational model of self-other integration via within- and between-person action-perception links, implemented as a simple Kuramoto model with four oscillators. The model abstracts each member of a dyad as a unit consisting of two connected oscillators, representing intrinsic processes of perception and action. By fitting this model to data from two separate experiments we show that interpersonal synchronization strategies rely on the relationship between within- and between-unit coupling. Specifically, *mutual adaptation* exhibits a higher between-unit coupling than within-unit coupling; *leading-following* requires that the follower unit has a low within-unit coupling; and *leading-leading* occurs when two units jointly exhibit a low between-unit coupling. These findings are consistent with the theory of interpersonal synchronization emerging through self-other integration mediated by processes of action-perception coupling. Hence, our results show that chaotic human behaviour occurring on a millisecond scale may be modelled using coupled oscillators.

## Author summary

Interacting with other people is crucial for our everyday life. We regularly and without much effort synchronize our movements with other people’s movements, across a wide range of interactions such as when dancing together and performing music. However, even a very simple interaction such as tapping a rhythm together can result in complex interaction dynamics. Two people tapping can choose different synchronization strategies, such as leading-following wherein one person is a leader and the other a follower, mutual adaptation where both adapt to each other, or leading-leading where both try to lead at the same time. In this paper we show how these strategies can be described using a mathematical model of coupled oscillators. Specifically, we show how the strategies rely on different coupling strengths between the oscillators in the model. This shows that even complex human behaviour may be modelled using simple mathematical terms.

## Introduction

**Fig 1:**
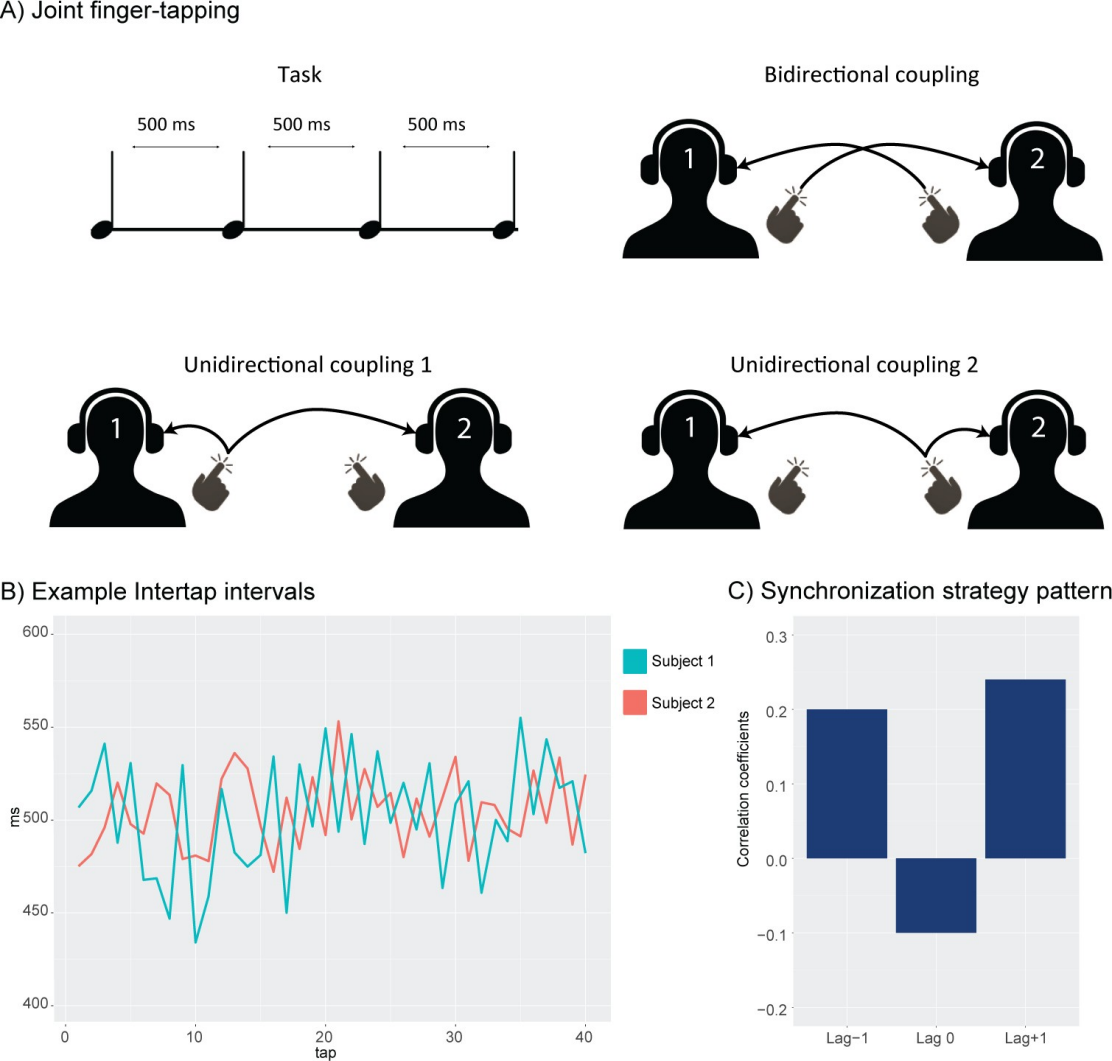
**In A) a joint finger tapping paradigm is illustrated. Two persons (dyad members) tap an isochronous rhythm together.** Their auditory feedback can be manipulated so that the dyad is bidirectionally coupled, i.e. that dyad member 1 hears dyad member 2 and vice versa, here illustrated in the top right. In the two bottom illustrations we see unidirectional coupling, wherein the information between dyad members only goes one way, for instance so that dyad member 1 only hears dyad member 2, and dyad member 2 hears only themselves. B) Time series representing the intertap interval, a measure of the time between successive taps, of each dyad member. Colours indicate dyad member. When these time series are cross-correlated at lag -1, lag 0, and lag +1, a pattern such as illustrated in C) emerges. Here, the pattern would indicate a mutual adaptation synchronization strategy.

When two people perform a simple task together, such as walking together or applauding after a successful performance, they tend towards synchronization [1, 2]. This emergence of synchronization is also found in many other natural phenomena [3], such as the collective flashings of fireflies [4], or the pacemaker cells in the heart [5]. For this reason, the mathematical framework of coupled oscillators provides an approach for understanding the conditions and parameters necessary for synchronization to emerge [6–8]. However, in many cases of human interaction, synchronization is not simply emergent, but rather a goal or a prerequisite of the task. A particularly prominent example of this is rhythmic joint action, as found in musical performance. Here, multiple people coordinate their movements and adapt to each other on a millisecond basis. In this sense, human interpersonal synchronization presents as a more complex system. Experiments using joint finger tapping paradigms (illustrated in Fig 1) show that this type of synchronization relies on different synchronization strategies, such as mutual adaptation and leading-following [9–12]. Common for these is that they cannot be differentiated by looking merely at measures of synchronization, as different strategies may exhibit the same synchronization level. Instead, differences between synchronization strategies can be detected when a lagged cross-correlation is calculated between the resultant time-series, as illustrated in Fig 1B and 1C [9]. Here, we see how a cross-correlation performed at lag -1, lag 0, and lag +1 gives rise to characteristic lag patterns, which are used to infer the synchronization strategy in use.

The most commonly found strategy is mutual adaptation, which occurs when both members in an interacting dyad simultaneously and continuously adapt to each other on a per-action basis [12, 13]. This results in positive correlations at lag -1 and lag +1, and usually a negative correlation at lag 0, as the members are mutually correlated with the previous tap of the other. Another well-documented synchronization strategy is the leading-following strategy, where one of the dyad members exhibits less adaptability than the other and hence becomes a leader. This results in a positive correlation at lag +1 (or lag -1, depending on which member is leading), and no correlation at lag -1 (or lag +1). While both these strategies have been reported in multiple studies, recently a third strategy called leading-leading was found [1, 9, 10, 14]. In the leading-leading strategy both members resist adaptation and rather tap along without much regard to the performance of their tapping partner. This results in a pattern with low correlation coefficients across all lags. While there have been attempts at modelling such behaviour with coupled oscillators, the existing models have not focused on capturing the mechanisms underlying these distinct synchronization strategies [1, 15–18].

One explanation for the emergence of these different strategies may lie in the increased complexity compared to physical systems such as coupled pendulums. In humans, the coupling is mediated by perceptual links, such as visual or auditory information, as opposed to the physical coupling found in non-biological systems [12]. Unsurprisingly, if no such link is present (for instance if one cannot perceive the other’s movement) experiments show that synchronization does not occur [19]. Hence, interpersonal synchronization necessitates two separate processes, wherein one process is perceiving the stimuli to be synchronized to, and another process is in charge of producing the actions leading to synchronization. The last couple of decades of research point towards these two processes, action and perception, being intrinsically coupled in terms of processing in the human brain [20, 21]. In joint finger tapping such action-perception coupling can occur when one dyad member perceives the auditory feedback from the other member as belonging to its own tapping, hence blurring the lines between self and other. It is this type of coupling that has been hypothesized to underlie the mutual adaptation synchronization strategy observed in previous studies [12]. On the other hand, if one dyad member chooses to ignore feedback from the other dyad member and instead solely monitors its own model of the task, this will force the other dyad member to take over the coordination task, and thus create a leading-following relationship. By necessity, this then requires the leading dyad member to decouple its motor actions from its auditory perception of the other.

Here we test the hypothesis that synchronization strategies emerge as a function of action-perception coupling strength. Specifically, we test if the rhythmic joint finger tapping tasks commonly used in the field of joint action can be modelled using a coupled oscillator model, and if empirically encountered synchronization strategies are systematically linked to differing coupling strengths in the model. This type of computational modelling method is a powerful approach towards elucidating the rules governing the behaviour of coupled dynamical systems. Specifically, the joint finger tapping tasks modelled in this paper illustrate social interaction on a millisecond scale, and insight into its universal rules may translate into a better understanding of psychiatric disorders involving atypical social interaction, such as autism and schizophrenia [22–24]. In the first part of this study, we examine the behaviour of a four-oscillator Kuramoto model to determine its ability to reach and maintain synchronization within the short amount of time as is seen in joint finger tapping experiments, while at the same time producing distinct synchronization strategies. We chose this type of continuously coupled model for its well-documented behaviour, previous application to interpersonal synchronization tasks, and generalized application to a broad range of synchronization phenomena observed in nature [3, 15, 18, 25–27]. In the second part we use empirical data to validate the model, and determine how coupling parameters are linked to specific synchronization strategies.

**Fig 2:**
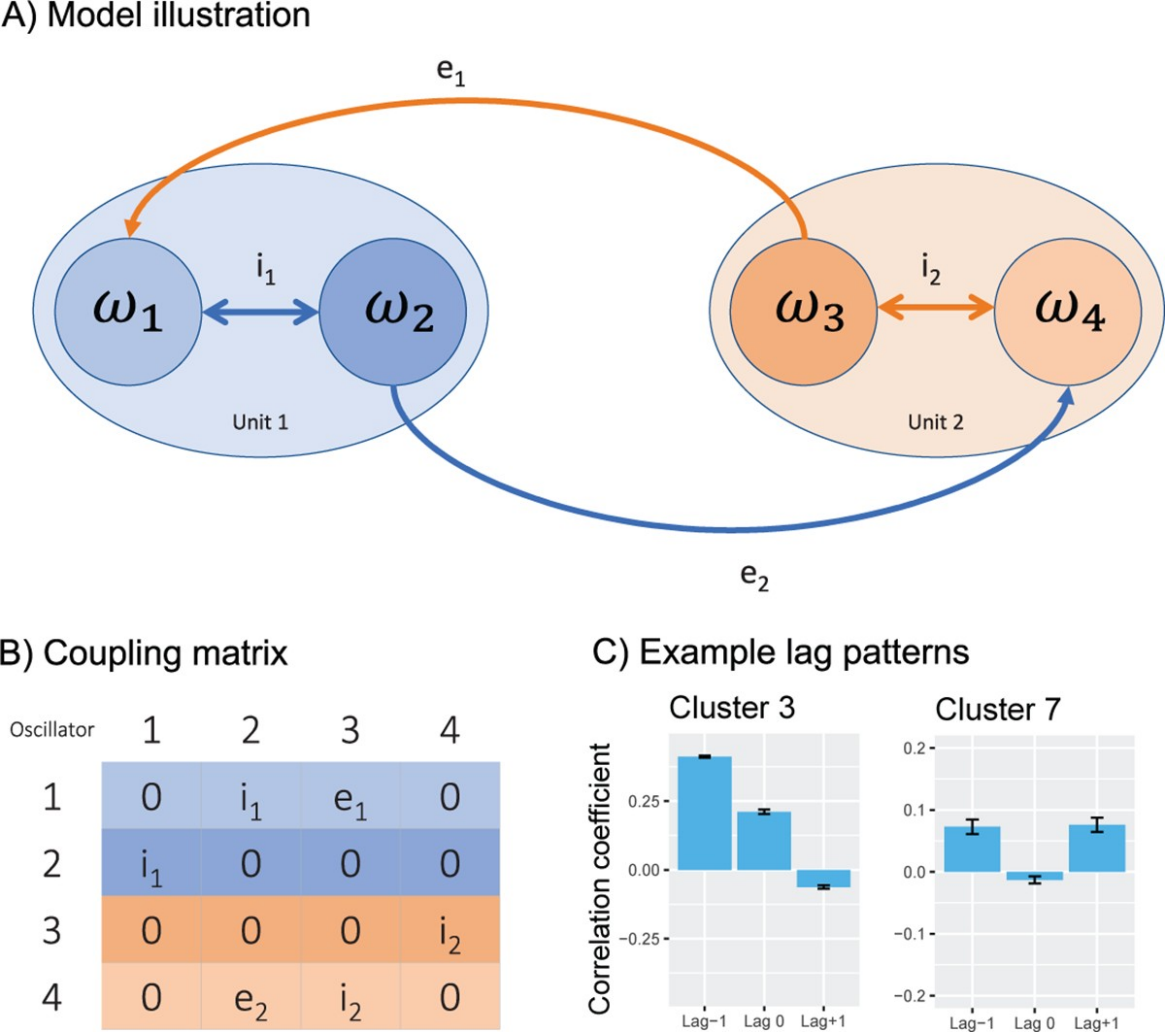
Overview of the model. In A we see the four-oscillator model, with the oscillators represented as circles within the two units. The coupling terms are shown as arrowed lines. In B the coupling matrix K_np_ is shown, and two out of 12 significantly different lag patterns produced by the model are shown in C.

Our model aims at representing the dynamics of interacting dyads performing joint finger tapping in a reduced form, retaining only the necessary features to capture the fundamental principles underlying the complex synchronization strategies observed in joint finger tapping. Each person is considered a unit, with two internal oscillators serving as proxies for perception and action (see Fig 2A). These two within-unit oscillators are bidirectionally linked through the internal coupling term *i* representing the intrinsic coupling found between auditory and motor processes in the brain. The two units are coupled so that the action oscillator in one unit is unidirectionally linked to the perception oscillator in the other unit, through the external coupling term *e*. This coupling term represent the extrinsic flow of information between the two interacting units. The model is based on the Kuramoto model of coupled oscillators, with the exception that the connection strength between each pair of oscillators *n* and *p* is defined by the coupling matrix *K*_*np*_ (Fig 2B), as defined by the following equation:

$$\frac{{d\theta }_{n}(t)}{dt}=\omega_{n}(t)+\sum_{p=1}^{k}K_{np}\;\sin\left(\theta_{p}(t)-\theta_{n}(t))+\xi (t\right),\;n=1,..,k$$ (1)

where *θ*_*n*_ is the phase of each oscillator *n*, *ω*_*n*_ is its intrinsic frequency, and *ξ* is a Gaussian noise component with mean *μ* = 0 and standard deviation *σ*. Together, the variability in frequency and the added noise represents the natural variability and noisiness in joint finger tapping (see full details in the Methods section) [27, 28]. It is important to note that a continuously coupled model such as this does not accurately capture all the aspects of information flow in a joint finger tapping task. For instance, the auditory information between dyads is transmitted in short bursts rather than continuously as in this model. In addition, it is likely that individual differences in variables such as multimodal feature integration and perspective taking also impacts the dynamics of interacting dyads [10, 29]. However, here we aimed at creating a model as mathematically simple as possible, yet retaining explanatory power in relation to the synchronization strategies found in joint finger tapping, and in particular the balance between the lag +1 and lag -1 measures.

To examine the behaviour of the model we first determine the coupling strength producing maximum synchronization, by globally varying the four coupling terms (i_1_, i_2_, e_1_, e_2_) equally (for further details see the Methods section). This allowed us to determine the range of coupling strengths for which the model switches from exhibiting unsynchronized to fully synchronized behaviour. Following this, we sampled the model at a selection of coupling strength combinations, and calculated cross-correlation lag patterns of the output from each unit’s action oscillator (ω_2_ and ω_3_ in Fig 2A). These lag patterns were thereafter clustered to identify significantly different lag patterns.

## Results

Simulations showed that the model reached a maximum synchronous state at an equal coupling weight of 15.5 1/s on all coupling parameters, as measured with the synchronization index [30]. This index is calculated based on the variance of relative phase between two signals, and is a unitless number ranging from 0 to 1, with 1 indicating full synchronization. Subsequently, we ran further simulations for a combination of selected coupling weights (see Methods) below this critical range to determine if the model was able to produce distinct lag patterns that are consistent with empirically observed synchronization strategies. We found that the model exhibited a rich and varied sample of lag patterns, with 12 of these patterns being different at α<0.001. Multiple clusters produced by the model have patterns with resemblances to a leading-following strategy (see Cluster 3 in Fig 2C) and one cluster exhibits features of a mutual adaptation strategy (see Cluster 7 in Fig 2C). Hence, we found that a model with four coupled oscillators and four coupling terms are able to produce an array of differing lag patterns, with some sharing similarity to known synchronization strategies.

To verify if this behaviour was dependent on each unit having two oscillators, we tested an even more reduced model containing only one oscillator per unit. This reduced model failed at producing lag patterns consistent with synchronization strategies, by showing either a considerable positive lag 0 component, or weak correlations across the lags (see methods).

To validate our model, we tested it on empirical data from two separate joint finger tapping studies. Dataset 1 was acquired from a 2018 study by Heggli et. al [14], and dataset 2 was acquired from the 2010 study by Konvalinka et. al [9]. Both datasets were collected in compliance with local and national research ethics standards.

In dataset 1, musicians were paired and asked to tap one of two rhythms together while bidirectionally coupled. Both rhythms had an intertap interval of 500 ms, corresponding to a beat-per-minute (bpm) of 120. A cluster-analysis of the cross-correlated lags identified three subgroups of participants. One subgroup used the leading-leading strategy, and the two remaining subgroups exhibited patterns of mutual adaptation at two different cross-correlation coefficient strengths.

In dataset 2, pairs of non-musicians tapped together in differing auditory coupling conditions: 1) uncoupled, with no auditory feedback from the other dyad member, 2) unidirectional coupling so that dyad member 1 hears their own tapping sound and member 2 hears member 1’s tapping, and mirrored so that member 2 hears their own tapping and member 1 hears member 2’s tapping, and 3) bidirectional coupling wherein dyad member 1 only hears the taps of dyad member 2 and vice versa. In addition, the tapping was performed at different tempos (96 bpm, 120 bpm, and 150 bpm). Here, a leading-following was found in the unidirectional condition, and mutual adaptation in the bidirectional condition. For the purposes of model validation, we chose only the 120-bpm tempo from this dataset, and only the conditions wherein the dyad members interacted (unidirectional coupling, and bidirectional coupling). Together, these two datasets contain three distinct synchronization strategies, in six independent groups.

We performed a two-step consecutive parameter search to determine which coupling weights best fit the data. For dataset 1 we used the three subgroups found in the empirical data, and for dataset 2 we used data from three different coupling conditions (bidirectional coupling, unidirectional coupling 1 with dyad member 1 being the leader, and unidirectional coupling 2 with dyad member 2 being the leader). We optimized our search on the numerical distance between the averaged cross-correlated lags in the empirical data and the data produced by our model. Once the best fit was found, we calculated the mean Bhattacharyya coefficient between the empirical and simulated data to serve as a measure of goodness of fit [31].

**Fig 3:**
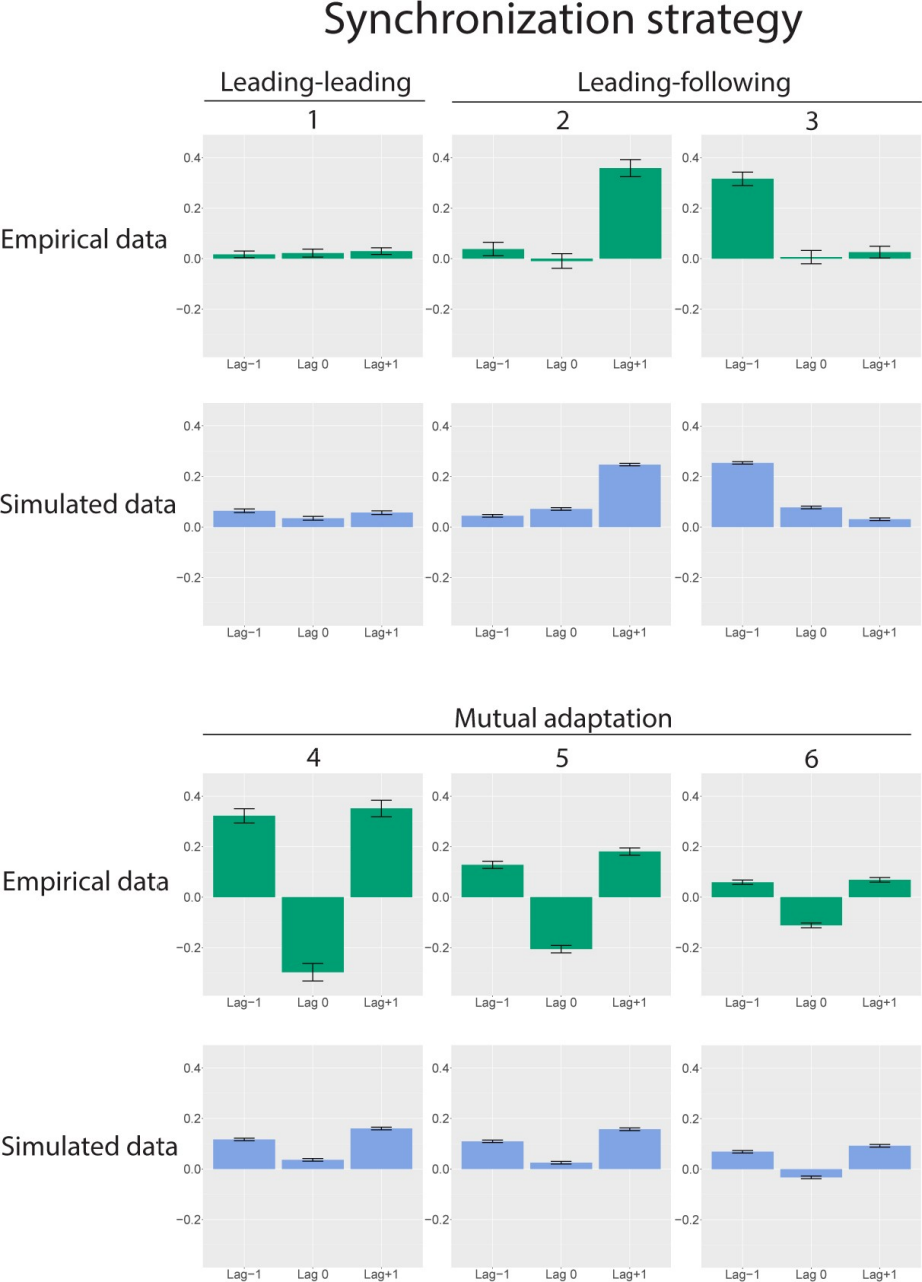
Overview of the main results. In the first row, synchronization patterns from the empirical data are shown. Leading-leading (1) corresponds to a subgroup from dataset 1. Leading-following (2) and (3) are from the two unidirectional conditions in dataset 2. From the mutual adaptation group, (4) is the bidirectional condition from dataset 2, whereas (5) and 6) are the two remaining subgroups from dataset 1. The green lag patterns show the empirical data. The blue lag patterns show the synchronization strategy patterns produced by the model at the given coupling weights listed in Table 1. The patterns are plotted as the mean value, with error bars indicating the standard error of the mean. Note that the lag 0 component in the simulated data is consistently more positive than in the empirical data, likely as a result of the continuous coupling in the model.

Our parameter search provided a good fit for all subgroups found in the empirical data (see Fig 3). We found that the three empirically encountered synchronization strategies, as reproduced by our model, are characterized by the weighting of between- and within-unit couplings. Mutual adaptation, the most common synchronization strategy, relies on a high between-unit coupling strength and a correspondingly low within-unit coupling strength. Leading-following, in our case the unidirectionally forced type of leading-following, relies on the leader unit having a balanced coupling strength on both the within- and between-unit coupling term. However, the follower unit exhibits a much stronger between-unit coupling strength than its within-unit coupling. The remaining strategy, leading-leading, presents as both units having a strong within-unit coupling strength, and a low between-unit coupling strength. The weakest component of the fit between empirical and simulated data is the lag 0 component, which becomes apparent in the instances of mutual adaption. This may stem from the continuous coupling used in our model, where the units share information in continuous time as opposed to in discrete taps as found in the experimental data. Nonetheless, the mean Bhattacharyya coefficients were over 0.8 in all cases except for one instance of the mutual adaptation strategy found in in dataset 2 (see Table 1).

**Table 1:** Overview of the best coupling weights found for each group. The numbering of the groups corresponds to the labelling used in *Fig 3*. The Bhattacharyya coefficient listed here is the mean coefficient between the three lags. The coupling strengths i1, e1, i2, e2, from the coupling matrix K are measured in 1/s and scaled according to the integration time step used in the simulations. For details, see Methods section.

Overview of best coupling weights	Unit 1		Unit 2		
*Coupling term*	i_1_	e_1_	i_2_	e_2_	Bhattacharyya coefficient (mean)
*1. Dataset 1—Leading-leading*	6.5	1.5	7.8	1.3	.97
*2. Dataset 2—Leading-following (1)*	1.7	5.5	4.1	5.5	.82
*3. Dataset 2—Leading-following (2)*	4.1	5.7	1.7	4.5	.81
*4. Dataset 2—Mutual adaptation (1)*	2.5	6.3	2.3	5.1	.77
*5. Dataset 1—Mutual adaptation (2)*	2.5	4	2.3	8	.93
*6. Dataset 1—Mutual adaptation (3)*	1	5	1.3	3.3	.98

## Discussion

Our results show that complex human behaviour can be described by a reduced model consisting of four oscillators and four coupling terms. While there have been multiple previous attempts at modelling interpersonal synchronization using either an information-processing or a dynamical systems approach, our work is the first to reproduce all three empirically-observed synchronization strategies [1, 15–18]. We find that these strategies rely on the balance of within- and between-unit coupling strengths in our model, and are placed at different points in the parameter space of the model. Mutual adaptation is found when the interacting units symmetrically downregulate their within-unit coupling strength with a corresponding increase in the between-unit coupling strength. Leading-leading is found on the opposite side of this symmetric axis, with both units exhibiting a higher within-unit coupling strength than the between-unit coupling strength. We found that the leading-following synchronization strategy requires two asymmetric units, with the follower unit having a strong between-unit coupling and the leading unit a balanced between- and within-unit coupling. If we consider the unit’s oscillators to represent processes of auditory perception and motor action our findings are consistent with theories positing that synchronization strategies emerge as functions of action-perception coupling [12].

Given these results, mutual adaptation in bidirectionally coupled joint finger tapping can be seen as a form of self-other merging, whereby two interacting people collectively attribute the auditory feedback stemming from their tapping partner as intrinsically linked to their own tapping actions. This results in an interaction wherein both dyads continuously and reciprocally adapt their tapping to each other on a tap-to-tap basis. In this sense, the dyad members can be considered to actively and collectively work towards minimizing the difference between their action and the related auditory feedback, resulting in a strong interpersonal action-perception coupling. This leads to the dynamics of the oscillators being predominantly governed by the information flow between the units, as expected in mutual adaptation. In the leading-leading strategy, this relationship is reversed.

The most likely behavioural explanation of leading-leading is that the dyad members both decouple the self-other loop, and instead focus on their own representation of the task [14]. Accordingly, our best fit for this strategy shows that both units exhibit a strong within-unit coupling, and a weak between-unit coupling. Hence, information flowing between the two units does not, to a noticeable degree, impact the behaviour of the individual unit. A key factor in understanding the emergence of this synchronization strategy is that in the behavioural experiment the two participants are in fact strongly coupled to the same external metronome at the start of the task. In our model, this is reflected in the starting frequency of the oscillators, and given a strong enough within-unit coupling this frequency is preserved to the point where synchronization occurs without the need for a strong between-unit coupling to modulate any deviance in the starting frequency. Hence, given two participants with sufficient skill it is possible to exhibit synchronization, predominantly as an artefact of their beat-keeping skills. However, the small, but non-zero, between-unit coupling may then function as an error-detection threshold, such that if one participant should strongly deviate the other may still choose to follow.

For the leading-following strategy wherein the leader hears themselves and the follower only hears the leader, the model converges on the leading unit having a balanced weight on its within- and between-unit coupling. The follower unit exhibits a stronger self-other (between-unit) coupling than its within-unit coupling. To achieve synchronization, the follower needs to consistently monitor the auditory feedback coming from the leader, while the leader is decoupled from the follower. It is interesting that the model here converges to a balanced within- and between-unit coupling in the leading unit. One likely explanation for this comes from the characteristics of the participants in dataset 2, which were all non-musicians. This is evident in the increased noisiness of their tapping, and we reflected this in the simulations by having an increased noise level as compared to the simulations for dataset 1 (see Methods). Having a balanced within-unit coupling may then act as a sort of self-correcting behaviour, ensuring that the contribution from noise in the individual oscillator is kept from increasing to heavily. It should also be noted that the leading-following behaviour seen here is experimentally forced due to the unidirectional auditory coupling between the participants. Previous research has shown that the leading-following strategy can also occur in cases of bidirectional auditory coupling, and that leaders can be distinguished from followers by increased frontal alpha suppression measured with EEG [11]. This finding has been interpreted to indicate an increase in cognitive load for leaders in bidirectionally coupled leading-following, due to the need for separating the auditory information from the followers from their own tapping actions. In this case, it may be that unidirectional leading-following differs from bidirectional leading-following, and it remains a possibility that our model would choose differing coupling weights for this strategy dependent on the participant’s auditory feedback.

The weakest point in our model appears to be the lag 0 component in the mutual adaptation strategy, as is evident from the low measure of fit found in the bidirectional condition in dataset 2. A likely explanation for this is that our model is continuously coupled, and is therefore able to adjust also when there would be no information present in a real-world setting, such as between taps. A solution for this would be to couple the two units intermittently, so that the coupling only exists when one unit produces an output. Such type of pulse-coupled oscillators have previously been used, for example, to model groups clapping in unison, and a hybrid approach may improve the fit of our model [1, 32]. In addition, due to limitations in the perceptual threshold, we would suggest including a filtering method wherein such information would only be passed between the units if it exceeds a pre-defined tolerance region, akin to mechanisms of predictive coding [33]. Another avenue for improvement could be implementing a feature pulling the action oscillator towards the participants’ preferred tapping rate [34]. For future work we would therefore primarily suggest that incorporating time varying coupling weights could prove beneficial towards modelling human behaviour with coupled oscillators.

As we have discussed above, all three synchronization strategies can be interpreted as emerging from different combinations and strengths of within- and between-person action-perception links. A likely explanation for how and why such action-perception links emerge can be found in mechanisms of self-other integration. There is ample evidence that the human brain processes perceived and performed actions using overlapping networks (for a review see Keysers and Gazzola 2009) [35]. For instance, observing an action can produce activity in motor areas of the brain, and observing someone else being touched can lead to activity in somatosensory regions in the brain [36]. Hence, there needs to be a mechanism that distinguishes between actions related to the self, and to others, commonly referred to as self-other representation. This refers to the process of categorizing whether a percept belongs to self- or other-produced actions. One way of considering action-perception coupling would then be that it occurs as the result of minimizing the distance between self- and other-representations. In other words, in tapping tasks such as those used in this work, action-perception coupling may stem from participants categorizing the auditory feedback they hear as related to their own tapping actions, instead of belonging to their tapping partner. This view of action-perception coupling finds support in the brain’s tendency towards minimizing computing costs, as formalized by Friston’s work on the free energy principle and its implications for theory of mind [12, 37–40]. Here, the brain is considered to constantly strive for energy optimized representations of its environment. In joint finger tapping, minimizing the difference in self-other representation decreases the need for maintaining a cognitive model of the tapping partner’s behaviour. This would suggest that mutual adaptation is a strong attractor state, particularly in interactions where there is information symmetry such as in most joint finger tapping studies [12]. However, mutual adaptation is not the only stable synchronization strategy in interpersonal synchronization as both leading-following and leading-leading have been shown to emerge in cases of bidirectional coupling [11].

**Fig 4:**
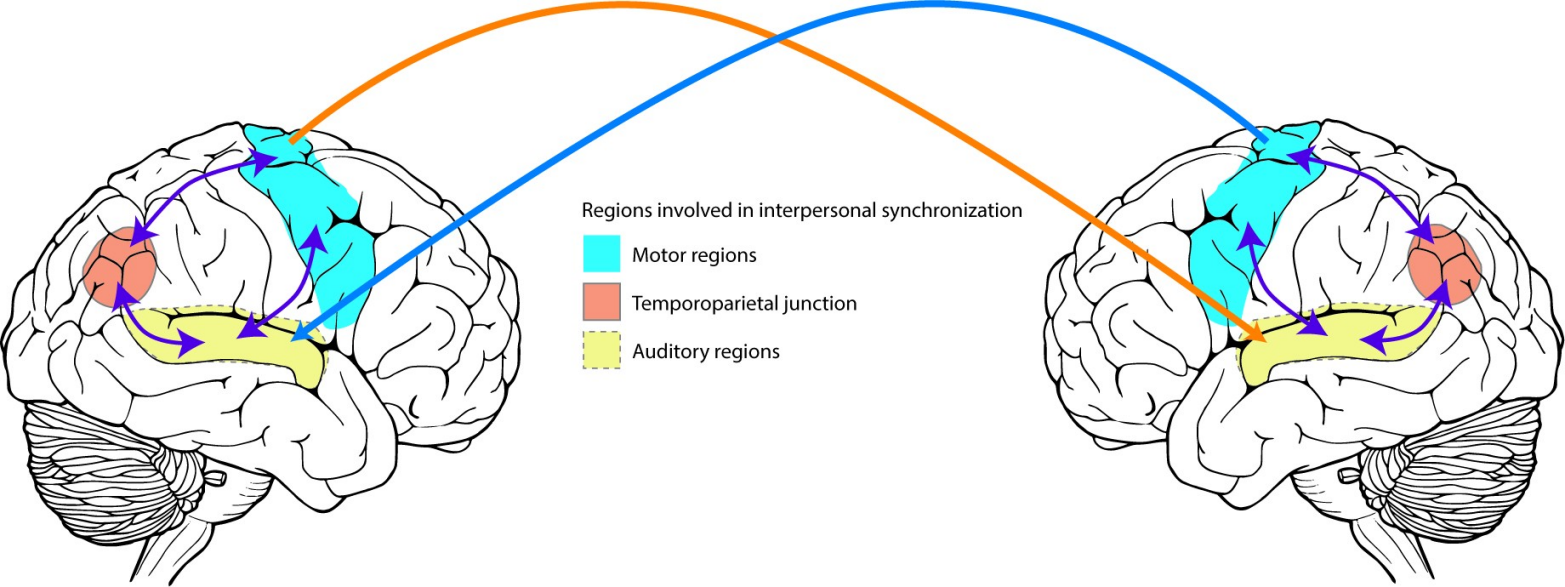
Illustration of regions involved in interpersonal synchronization. Motor regions (shown in light blue) are bidirectionally linked to auditory regions (shown in light yellow), and with the temporoparietal junction (shown in light red). Actions produced by one individual’s motor system is perceived in auditory regions of the other individual.

As with any computational model of a complex real-world process, our model can only approximate the processes involved. When considering the neural underpinnings of interpersonal synchronization, our model does not make any strict assumption. Rather, the structure of the model may be interpreted to represent action-perception coupling, as part of the more complex processes of self-other representation. Likely, these processes are all involved in interpersonal synchronization and in the selection of synchronization strategies. As rhythmic interpersonal synchronization requires auditory perception, and motor action, we expect brain regions and networks linked to such to be involved. It is also likely that regions and networks linked to social cognition are involved. For instance, our data includes a case of bidirectional coupling resulting in two distinctly different synchronization strategies, mutual adaptation and leading-leading. Previous research has also shown the existence of leading-leading in cases of bidirectional auditory coupling [11]. Hence, there needs to be a neural structure or network involved in the selection of synchronization strategy, that is independent of auditory coupling. A likely candidate here is the temporoparietal junction (TPJ) [41]. This region, located where the temporal and parietal lobes meet, has been shown to act as a network node between the thalamus and the limbic system as well as with sensory systems [42], and is proposed to be a key region in brain networks implicated with ‘mentalizing’ [22]. In particular, the right TPJ is involved in segregating self-produced actions from actions produced by others, as is shown in lesion studies and in studies using transcranial magnetic stimulation [43, 44]. We would therefore hypothesize that the involvement of the TPJ, either separately or as part of a distributed network, is a key factor in the emergence of synchronization strategies [45]. An interpretation of our model is then that the two oscillators represent an interplay between auditory and motor regions mediated by the TPJ, as shown in Fig 4. Here, the between-brain couplings rely on the auditory perception of the motor actions produced by the other. We hypothesize that synchronization strategies may be distinguished in electrophysiological recordings by activity in such a network. For instance, mutual adaptation likely requires neural synchronization between representation of self- and other, as mediated in tapping tasks by motor and auditory systems in the brain [12]. Hence, one would expect to see more coherent activity between the involved brain regions during mutual adaptation than in leading-leading or leading-following.

## Conclusion

In this paper we have shown how synchronization strategies found in human joint action may be successfully modelled using a reduced model consisting of four oscillators and four coupling terms. We found that synchronization strategies can be distinguished based on their within- and between-unit coupling strengths. For this particular study we interpret our model within the framework of self-other integration via within- and between-person action-perception links. However, we believe the model may be successfully applied to many other types of behaviour, such as modelling groups of people and other processes relying on perceptually mediated couplings between individuals. Here, an interesting avenue for future research is predicting social behaviour in population groups with psychiatric conditions impeding self-other distinction, such as schizophrenia [46]. As schizophrenia is linked with reduced activity in the TPJ, a comparison of patients with schizophrenia with a neurotypical population could offer a way to test predictions made by our model as well as our hypothesized neural interpretation shown in Fig 4 [47, 48].

The coupled oscillators approach has previously been successfully used for modelling synchronization behaviour in group interactions, and a logical next step for future work is comparing our two-oscillator unit approach with, for instance, the second order coupling term variation of the extended Haken-Kelso-Bunz model proposed by Zhang and colleagues [8, 27, 49, 50]. Our model is easily scalable, although increasing the interacting units comes at computational cost. In informal tests with up to 200 interacting units we observe complex behaviours such as short-lived stable states of both in- and anti-phase synchronization. Hence, the model may be a promising tool for exploring network topologies in multi-person interactions with natural occurrences of leading and following behaviour, such as for instance in symphony orchestras or in jazz bands. We furthermore present a likely neural interpretation of the model, where we suggest that interpersonal synchronization strategies may be represented as coherence in a network between auditory and motor regions, and the temporoparietal junction. Our model and its behaviour suggest that complex human behaviour may be reasonably modelled by simple interacting components, and that coupled oscillators are able to capture these dynamics.

## Methods

### Implementation of the models

We implemented the models in MATLAB R2016b [51], using a script for performing numerical integration of the Kuramoto model of coupled oscillators [52, 53]. The phase of an oscillator *n* at time *t*, denoted by *θ*_*n*_(*t*), behave according to the following dynamic equation:

$$\frac{{d\theta }_{n}(t)}{dt}=\omega_{n}(t)+\sum_{p=1}^{k}K_{np}\;\sin\left(\theta_{p}(t)-\theta_{n}(t))+\xi (t\right),\;n=1,..,k$$ (2)

The coupling is defined by the matrix *K*_*np*_. When we use a two-oscillator model, this matrix is given by:

$$K_{model1}=[\begin{array}{cc} e_{1} & 0\\ 0 & e_{2} \end{array}]$$ (3)

whereas when we use the full four-oscillator model, this matrix is given by:

$$K_{model2}=[\begin{array}{cccc} 0 & i_{1} & e_{1} & 0\\ i_{1} & 0 & 0 & 0\\ 0 & 0 & 0 & i_{2}\\ 0 & e_{2} & i_{2} & 0 \end{array}]$$ (4)

Both models have a noise component *ξ* drawn from a Gaussian distribution with mean *μ* = 0 and standard deviation *σ*. This was included to account for the natural variability and noisiness observed in empirical data. As shown in Eq 2, the coupling weights measured in 1/s are scaled according to the integration time dt, and thus remain constant independent of simulation time and temporal resolution. For study 1 and for dataset 1 in study 2 the standard deviation *σ* was set to 0.2513 rad, which is equivalent to 20 ms at a frequency of 2 Hz, corresponding to the interquartile range (IQR) of the intertap intervals observed in the empirical data used for study 2 [14]. For dataset 2 in study 2, the standard deviation of the noise was set to 0.4335 rad, which is equivalent to 34.5 ms at a frequency of 2 Hz, corresponding to the IQR of the intertap intervals in dataset 2. Hence, the noise in the model was based on the shared dyadic variability in tapping rates. In all simulations the oscillators intrinsic frequency *ω* was set to 2 Hz (12.57 rad/s), with a standard deviation of 0.2 Hz (1.26 rad/s). This distribution accounts for the natural variations in the ability to catch and lock on to the metronome frequency [27, 28]. The oscillators were initiated at random phases. For inspecting the model’s performance and behaviour, we calculated the phases of the oscillators in steps of 25 ms and sampled the phase at intervals of 500 ms for computational efficiency, and linearly interpolated the zero-crossing point as a basis for creating a time-series of tap events. From this time-series of tap-events we calculated the intertap intervals. However, for the comparison between empirical data and simulated data reported in the model validation section, we calculated and sampled the phases of the oscillators in steps of 10 ms for increased accuracy.

### Model behaviour

**Fig 5:**
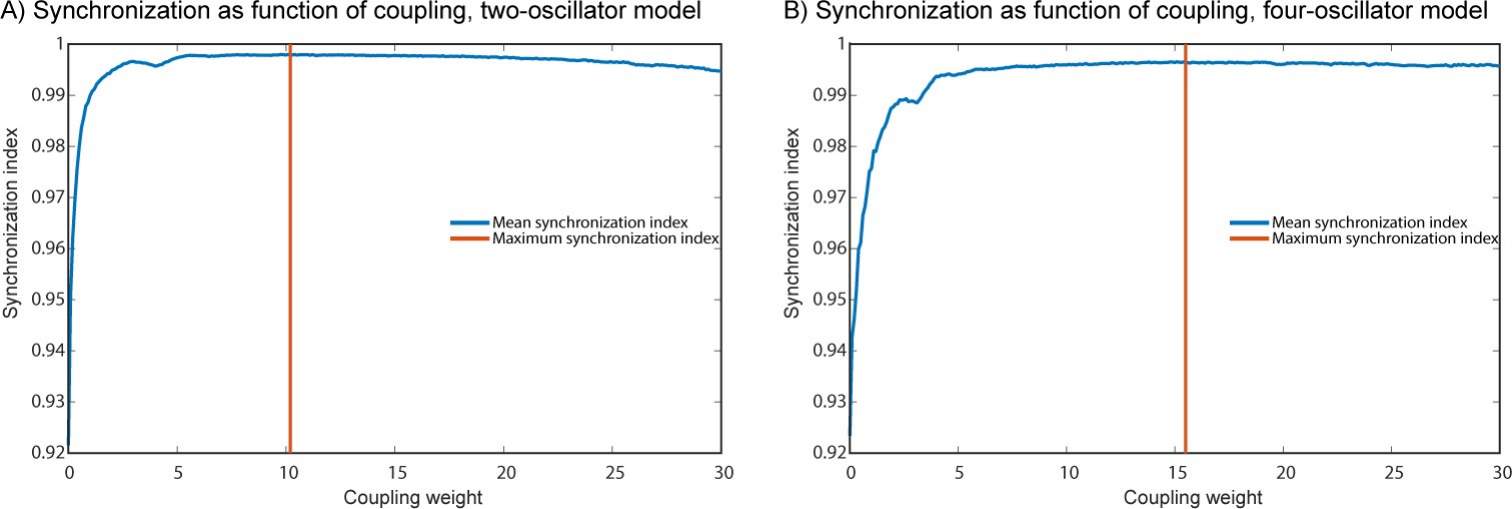
Synchronization as measured by the synchronization index as a function of coupling weight. In A we see the synchronization index of the two-oscillator model as a function of coupling weight. The vertical orange line indicates the point of maximum synchronization. In B the same is shown for the four-oscillator model.

To determine the range of coupling weights wherein the models transitioned from an incoherent to coherent state we ran simulations over a wider range of coupling weights. We linearly increased coupling weights equally for all coupling terms in both models, starting at a minimum coupling weight of 0.1 up to a maximum of 30 with a step size of 0.1. 200 simulations of 12 seconds each were averaged per step. Note that in model 2 we only considered synchronization between the two action oscillators. We found that the models quickly reached coherent synchronization state, as shown in Fig 5.

To examine the behaviour of the models we performed a parameter search within the previously determined bifurcation range. For the two-oscillator model, we restricted the search to a coupling weight of 0 to 10 in steps of 1 for between-unit coupling term 1 (*e*_1_), and let between-unit coupling term 2 (*e*_2_) range from *e*_1_ to 10, in steps of 1. This results in 66 possible combinations, and for each combination 200 simulations of 12 seconds were run. The oscillators were initiated at random phases, and the first two seconds of simulations were discarded to account for the metronome that was present in the first two seconds of the interaction in the empirical experiments. For the four-oscillator model we decided to sample the model at four different coupling weights, 1, 5, 9, and 13. This gives 256 possible combinations, which we simulated in the same way as the two-oscillator model.

**Fig 6:**
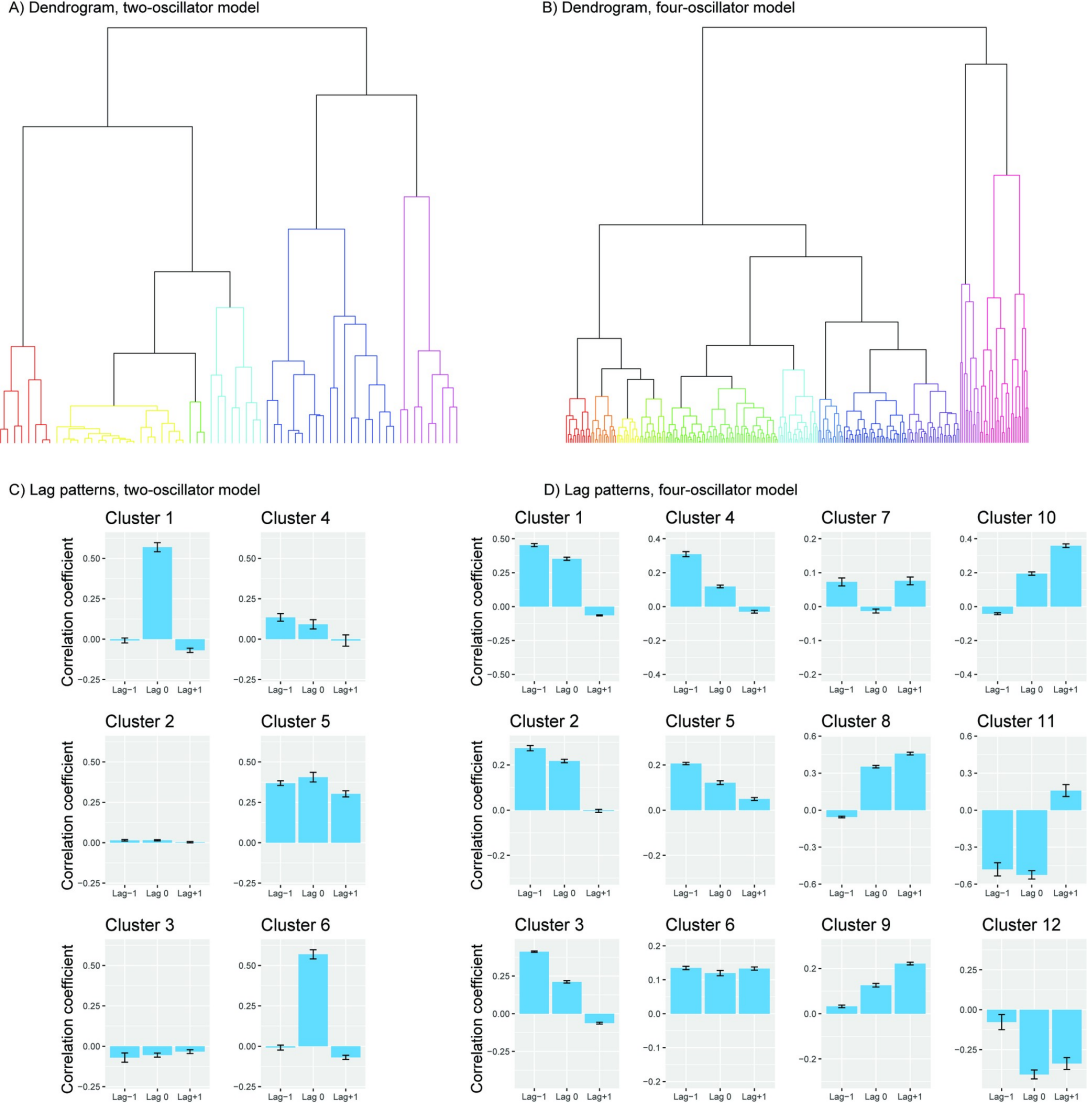
Clustering dendrogram and resulting lag patterns. In A and in B the clustering dendrogram for, respectively, the two-oscillator model and the four-oscillator model are shown. In C the corresponding mean lag patterns for the two-oscillator model is shown. In this case, the two-oscillator model produced six significantly different lag patterns. Out of these six, three exhibit a strong lag-0 component (clusters 1, 5, and 6), and the remaining three shows weak correlations across the lags. In D we show the same procedure applied to data from the four-oscillator model. Here we see a much richer variety of lag patterns, with 12 being significantly different. Note that the lag patterns produced from the clustering algorithm do not necessarily constitute synchronization strategies, but rather patterns that are quantitatively different from each other. The high number of different clusters also, in part, stems from the flip symmetry in the system, as is seen for instance in cluster 1 and cluster 8.

We analysed the time series produced by the model by performing a cross-correlation at lag -1, 0 and +1 for each simulation run. These correlations coefficients were then averaged for each coupling weight combination. We then clustered the lagged cross-correlations using the complete linkage method, and performed a similarity profile analysis in R [54], using the simprof-package [55], at an adjusted alpha of 0.001 (shown in Fig 6)

### Model validation on empirical data

To calculate the coupling weight which best fitted to the empirical data we performed a two-step consecutive parameter search. For both datasets we searched for the coupling weights that resulted in the best fit with each of the three subgroups of participants in the empirical data. For each of the three separate subgroups we first simulated 300 trials for each possible coupling value combination between the four oscillators, with the coupling weights ranging from 1 to 15 in steps of 1. Each trial was simulated using the same approach as for study 1. We averaged the cross-correlated lags of the individual time series produced in each trial per coupling value combination, and calculated the numerical distance between the simulated data and the empirical data. The best fit was then selected, and a second search performed at coupling weights of +- 0.9 in steps of 0.2 following the same procedure. The resultant best fit based on numerical distance was then chosen, and its coupling weights were used to simulate 2000 trials. Here, we increased the accuracy in the time series data by calculating and sampling the phase of the oscillators in steps of 10 ms. The Bhattacharyya coefficient was calculated for each of the three lags (-1, 0, and +1) between the simulated data and the empirical data separately and then averaged, using the disparity package in R [56]. While this two-step semi-exhaustive search may not be computationally optimal, it serves well when dealing with a noisy dataset and a noisy model. In particular, as we optimised on the cross-correlation lag coefficients calculated from the time series of phases generated by the model, existing algorithmic approaches such as the SINDy algorithm were not easily adaptable to our needs [57]. Our approach resulted in approximately 8 × 10^11^ simulations, which were performed over the course of roughly 60 hours on a MATLAB Distributed Computing Server.
